# Zhang’s guideline vs. 1994 WHO partograph: comparative effectiveness in managing hypertensive disorder of pregnancy

**DOI:** 10.1186/s12884-025-08441-y

**Published:** 2025-12-08

**Authors:** Cheng-Juan Sun, Yuanyuan Zheng, Shaofei Su, Jing Liu, Wei Song, Haili Jiang

**Affiliations:** 1 Department of Obstetrics, Beijing Obstetrics and Gynecology Hospital, Beijing Maternal and Child Health Care Hospital, Capital Medical University, No. 251 Yaojiayuan Road, Beijing, 100026 China; 2 Department of Central Laboratory, Beijing Obstetrics and Gynecology Hospital, Capital Medical University, Beijing Maternal and Child Health Care Hospital, No. 251 Yaojiayuan Road, Beijing, 100026 China

**Keywords:** Hypertensive disorder of pregnancy, Zhang’s guideline, 1994 WHO partograph, Postpartum hemorrhage, Forceps-assisted delivery, Advanced maternal age, Intrapartum cesarean section

## Abstract

**Background:**

Zhang’s guideline and the 1994 WHO partograph are both used to monitor labor progress. Zhang’s guideline defines labor’s active phase as cervical dilation of 6 cm (vs. 4 cm in the 1994 WHO partograph) and emphasizes individualized care with extended labor observation. Conversely, the 1994 WHO partograph uses a standardized “action line” for earlier intervention. This study compared Zhang’s guideline and the1994 WHO partograph in managing hypertensive disorder of pregnancy (HDP), specifically evaluating labor interventions, maternal age disparities, and postpartum outcomes.

**Methods:**

This retrospective cohort study analyzed clinical data from 5806 nulliparous women with singleton full-term pregnancies who were diagnosed with HDP between 2010 and 2023. Participants were stratified into the 1994 WHO partograph (January 2010–August 2014, *N* = 2100) and Zhang’s guideline cohorts (September 2014–December 2023, *N* = 3706). The primary endpoints were the intrapartum cesarean and postpartum hemorrhage (PPH) rates. Secondary endpoints included the rates of labor intervention (oxytocin augmentation, artificial membrane rupture, and lateral episiotomy), forceps-assisted delivery, and neonatal asphyxia (5-min Apgar score < 7).

**Results:**

Zhang’s guideline significantly reduced the intrapartum cesarean (10.96% vs.13.33%, *P <* 0.0001) and labor intervention rates (16.06% vs.43.62%, *P <* 0.0001) but increased the rates of PPH (20.02% vs.11.24%, *P <* 0.0001) and forceps-assisted delivery (19.67% vs.7.90%, *P <* 0.0001). Zhang’s guideline group included a higher of advanced maternal age (AMA, ≥ 35 years) pregnancies (20.72% vs.9.24%, *P <* 0.0001) and assisted reproductive technology (ART) usage (6.99% vs.0.90%, *P <* 0.0001). The frequency of neonatal asphyxia did not differ between the groups. Multivariate analysis illustrated that the use of Zhang’s guideline (odds ratio [OR] = 2.101, *P <* 0.0001), prolonged labor (OR = 1.607, *P =* 0.0052), and intrapartum cesarean section (OR = 6.024, *P <* 0.0001) were independent risk factors for PPH.

**Conclusions:**

Compared with the 1994 WHO partograph, Zhang’s guideline for managing HDP effectively reduced intrapartum cesarean sections and labor interventions. Zhang’s guideline also proved more adaptable to pregnancies involving AMA and ART without increasing the risk of neonatal asphyxia. However, its implementation was associated with higher rates of PPH and forceps delivery. Notably, the protocol itself emerged as an independent risk factor for PPH.

## Introduction

Hypertensive disorder of pregnancy (HDP) is a leading global cause of maternal and neonatal morbidity and mortality, significantly influencing pregnancy outcomes [1]. HDP includes chronic hypertension, gestational hypertension, pre-eclampsia, eclampsia, and chronic hypertension with superimposed preeclampsia. The management strategies for HDP directly affect pregnancy outcomes [2]. Globally, HDP is associated with approximately 200,000 stillbirths annually. Limited data exist on the optimal timing of delivery for women with well-controlled hypertension. A large retrospective observational study involving 228,668 women with gestational hypertension found that delivery between 38 and 39 weeks of gestation offers the best balance between minimizing maternal and fetal risks of morbidity and mortality [3]. The WILL trial (ISRCTN77258279) further explored this by comparing delivery outcomes between 38 weeks 0 days and 38 weeks 3 days [4]. Timely detection and effective management of labor progression in HDP are critical for improving pregnancy outcomes [5].

The 1994 WHO partograph and Zhang’s guideline are two widely utilized tools for monitoring labor progress [6]. As a globally standardized labor management tool, the 1994 WHO partograph has significant advantages in reducing unnecessary interventions and improving maternal and neonatal outcomes [7]. Research indicates that the 1994 WHO partograph can effectively reduce PPH rate and the rate of conversion to cesarean section, especially in resource-limited areas [8]. However, the 1994 WHO partograph has relatively low flexibility in the management of older pregnant women and complicated pregnancies, and it might not fully meet individualized needs [9]. In addition, the use of the 1994 WHO partograph in patients with HDP remains controversial. Some studies suggest that its effectiveness in reducing interventions and improving maternal and neonatal outcomes is limited [10]. Zhang’s labor curves, based on data from pregnant individuals in the United States, propose extending the duration of labor before diagnosing labor dystocia. The labor curves note that cervical dilation progresses more slowly than previously expected before reaching 6 cm. In managing, Zhang’s guideline emphasizes individualized care, reducing unnecessary intrapartum interventions, and optimizing labor analgesia [11].

Research illustrated that Zhang’s guideline has high adaptability among older pregnant women and users of assisted reproductive technology (ART; refers to treatments and procedures that aim to achieve pregnancy), and it can effectively reduce the rate of intrapartum cesarean section (ICS). ICS is defined as the change from a planned vaginal delivery to a cesarean section during the intrapartum period because of maternal–fetal medical indications or abnormal labor progress. Some studies reported that Zhang’s guideline decreased ICS rates, whereas others found no significant impact [12–14].Additionally, certain trials observed an increase in ICS rates among nulliparous women [15]. Moreover, the labor intervention rate is significantly lower for Zhang’s guideline than for the 1994 WHO partograph, suggesting its potential to promote natural childbirth [16]. However, the performance of Zhang’s guideline in PPH management remains controversial, and some studies indicated that it can increase the risk of PPH [17]. Therefore, the overall effectiveness of Zhang’s guideline in the management of high-risk pregnancies requires further verification [18]. Zhang’s guideline, which is used in China, is primarily based on data from European and American populations, and the data included only a small proportion (< 5%) of Asian participants. Consequently, this guidance does not sufficiently account for the unique demographic characteristics, cultural backgrounds, physical traits, medical environments, and lifestyle habits of childbearing women in Asia. In response to this, the Obstetrics Section of the Obstetrics and Gynecology Branch of the Chinese Medical Association issued the “Expert Consensus on the Standards and Treatment of New Labor Procedures” in July 2014. This consensus was formulated by integrating the specific characteristics of the Chinese population with recommendations from other countries [19]. However, few studies directly compared Zhang’s guideline with the 1994 WHO partograph in the labor management of individuals with HDP. Moreover, the superiority and inferiority of the two tools in terms of reducing interventions and improving maternal outcomes remain unclear.

This study systematically compared the effectiveness of Zhang’s guideline and the 1994 WHO partograph in managing HDP among nulliparous Asian women using a decade of clinical data from 5806 cases of HDP. The primary endpoints specifically evaluated the differential effects of the two guidelines on the rate of ICS and risk profiles for PPH. Secondary endpoints included the rates of labor intervention (oxytocin use, artificial membrane rupture, and lateral episiotomy), forceps-assisted delivery, and neonatal asphyxia (5-min Apgar score < 7). These findings aim to inform risk-stratified protocol implementation in diverse clinical settings.

## Materials and methods

### Study design and populations

This retrospective cohort study was conducted at Beijing Obstetrics and Gynecology Hospital (BOGH), Capital Medical University, a tertiary referral center managing approximately 15,000 deliveries annually. The study population consisted of all childbirth cases that met the inclusion criteria at BOGH from January 2010 to December 2023.The study compared labor outcomes between two distinct management protocols for HDP: the 1994 WHO partograph (January 2010–August 2014) and Zhang’s guideline (September 2014–December 2023).

Nulliparous women with singleton pregnancies (≥ 37 weeks’ gestation) who were diagnosed with HDP as defined by ICD-10 codes (O11–O15) were targeted. Eligible cases included gestational hypertension (O13), preeclampsia with or without severe features (O14; defined as systolic blood pressure ≥ 160 mmHg, thrombocytopenia, or end-organ dysfunction), eclampsia (O15), and chronic hypertension with superimposed preeclampsia (O11) [20]. All participants had vertex-presenting fetuses, and the final cohort studied include nulliparous women with HDP and planned trial of labor.

Exclusion criteria were applied to minimize confounding from comorbidities and pregnancy complications. The maternal exclusion criteria comprised pre-existing cardiovascular disease (New York Heart Association class II–IV), chronic kidney disease (estimated glomerular filtration rate < 60 mL/min/1.73m^2^), and autoimmune disorders such as systemic lupus erythematosus or antiphospholipid syndrome. The pregnancy-related exclusion criteria included placental abnormalities (placenta previa or accreta), preterm premature rupture of membranes (< 37 weeks), and intrahepatic cholestasis of pregnancy (serum bile acid level of > 40 µmol/L). The fetal exclusion criteria encompassed major congenital anomalies (structural or chromosomal), intrauterine demise prior to labor, and extremes of birth weight (< 2000 g or > 4500 g).

**Fig. 1:**
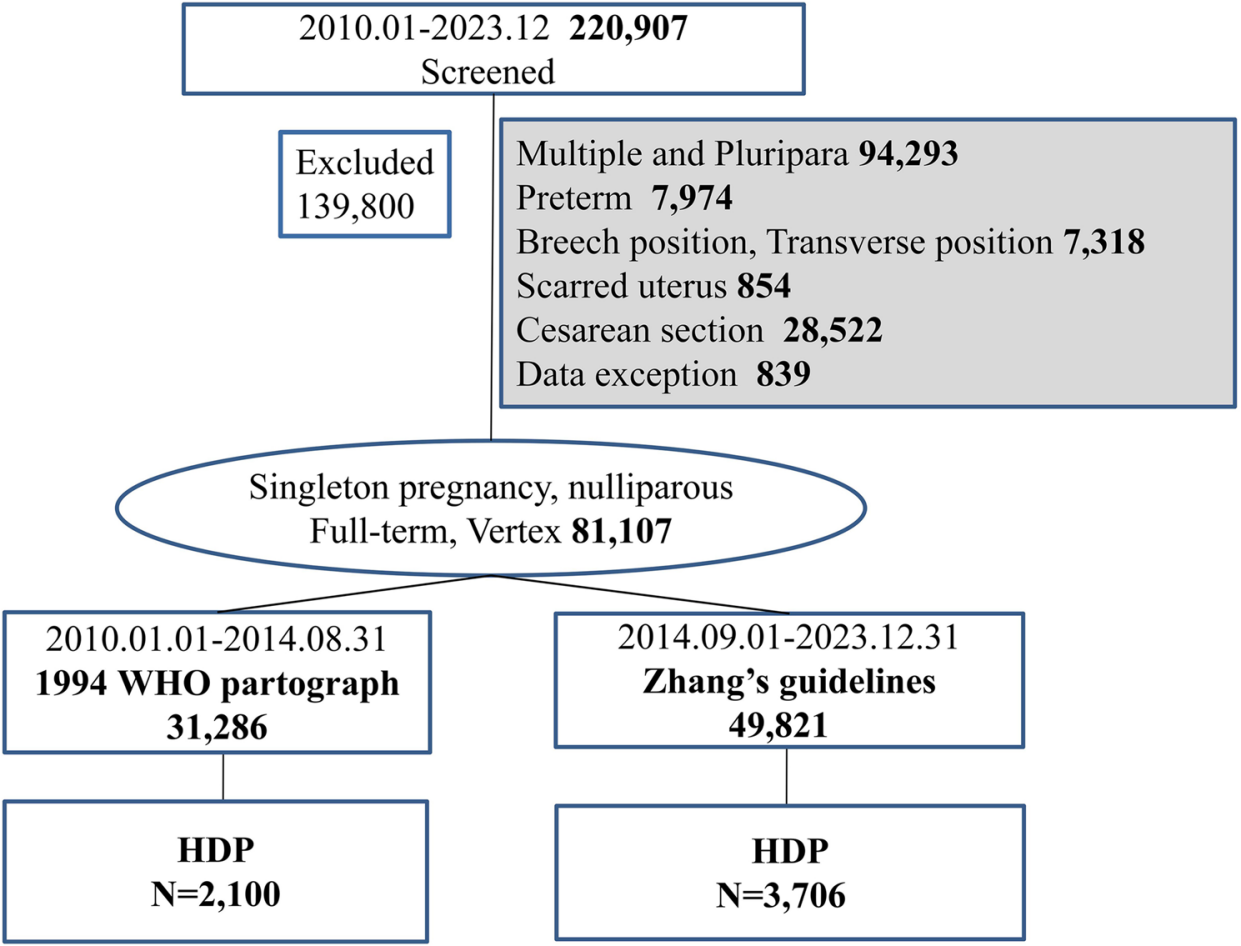
Study population selection and cohort allocation for full-term HDP under the 1994 WHO partograph and Zhang’s guideline. The left branch represents deliveries managed using the 1994 WHO partograph (January 2010–August 2014, *N* = 2100), whereas the right branch corresponds to those managed under Zhang’s guideline (September 2014–December 2023, *N* = 3706). Final cohort allocation reflects protocol-specific eligibility after rigorous screening

After rigorous application of these criteria, 5806 cases of HDP were retained for analysis, comprising 2100 women managed under the 1994 WHO partograph and 3706 women treated under Zhang’s guideline (Fig. 1). Baseline characteristics were strictly extracted from the electronic medical records of women with HDP who met the inclusion criteria, including maternal age, pre-pregnancy BMI, and HDP severity stratification.

### Labor management protocols

In the 1994 WHO partograph group, labor progression was monitored using the standard 1994 WHO partograph, initiating active phase documentation at cervical dilation of 4 cm. Interventions (e.g., oxytocin augmentation, cesarean section) were triggered when labor trajectories crossed the predefined “action line.” In Zhang’s guideline group, the guideline was implemented per the 2014 Chinese Expert Consensus on Labor Management. Active phase commencement was redefined at a dilation of 6 cm. Protracted labor was diagnosed if cervical dilation rates fell below 0.5 cm/h for ≥ 4 h, with oxytocin augmentation initiated at stricter thresholds. Labor intervention including oxytocin augmentation, artificial rupture of membranes, and lateral episiotomy.

Both groups received standardized HDP management, including hourly blood pressure monitoring, magnesium sulfate prophylaxis for severe preeclampsia, and timed delivery protocols per International Society for the Study of Hypertension in Pregnancy (ISSHP) guidelines.

### Data collection and variables

Well-trained obstetric doctors extracted data from patients’ electronic medical records using a validated case report form, capturing key variables across maternal characteristics, labor parameters, and delivery outcomes. Maternal characteristics included age (< 35 years vs. ≥35 years), pre-pregnancy body mass index (BMI, kg/m^2^), conception method (natural vs. ART), and comorbidities such as chronic hypertension and gestational diabetes. Regarding the diagnostic performance of pregnancy, obesity is defined as BMI ≥ 28 kg/m^2^ in China [21]. Labor parameters encompassed cervical dilation kinetics (latent and active phase durations), oxytocin administration (dosage and duration), and analgesia use. Delivery outcomes were categorized into primary and secondary endpoints. The primary outcomes were intrapartum cesarean section and PPH. PPH was uniformly defined as blood loss of ≥ 500 mL for vaginal deliveries or ≥ 1000 mL for cesarean deliveries. Blood loss was quantitatively measured using graduated under-buttock drapes, and this definition remained consistent throughout the entire study period. These condary outcomes comprised operative vaginal delivery (forceps), severe perineal trauma (third/fourth-degree tears), and neonatal asphyxia (5-min Apgar score < 7). To ensure data accuracy, 10% of records underwent blinded re-abstraction by senior obstetricians, achieving inter-rater reliability of κ = 0.89–0.93 for critical variables.

### Statistical analysis

Analyses were performed using SPSS 26.0 and R 4.2.2, with continuous variables compared *via* Student’s *t*-test or the Mann–Whitney U test and categorical variables analyzed using the chi-squared test or Fisher’s exact test. Multivariable logistic regression models adjusted for confounders, including Zhang’s or 1994 WHO guideline, advanced maternal age (AMA; ≥35 years), obesity, gestational diabetes mellitus (GDM), maternal age, assisted reproduction, labor duration of > 20 h, oxytocin augmentation, macrosomia, episiotomy, and delivery mode or gestational age at delivery. Temporal trends in intrapartum cesarean and PPH rates were evaluated using joinpoint regression to identify significant inflection points corresponding to protocol changes. Sensitivity analyses were conducted to address potential biases, including multiple imputation (50 iterations) for missing covariate data (< 5%), competing risk models (Fine–Gray subdistribution hazards) to account for censoring by emergent cesarean delivery, and E-value calculations to quantify potential unmeasured confounding.

### Ethical permission

The study protocol was approved by the Institutional Review Board of Beijing Obstetrics and Gynecology Hospital (No. 2024-KY-013-01) in compliance with the Declaration of Helsinki. The requirement for informed consent was waived for this retrospective analysis of de-identified data.

## Results

### Demographic characteristics and obstetric outcomes

**Fig. 2:**
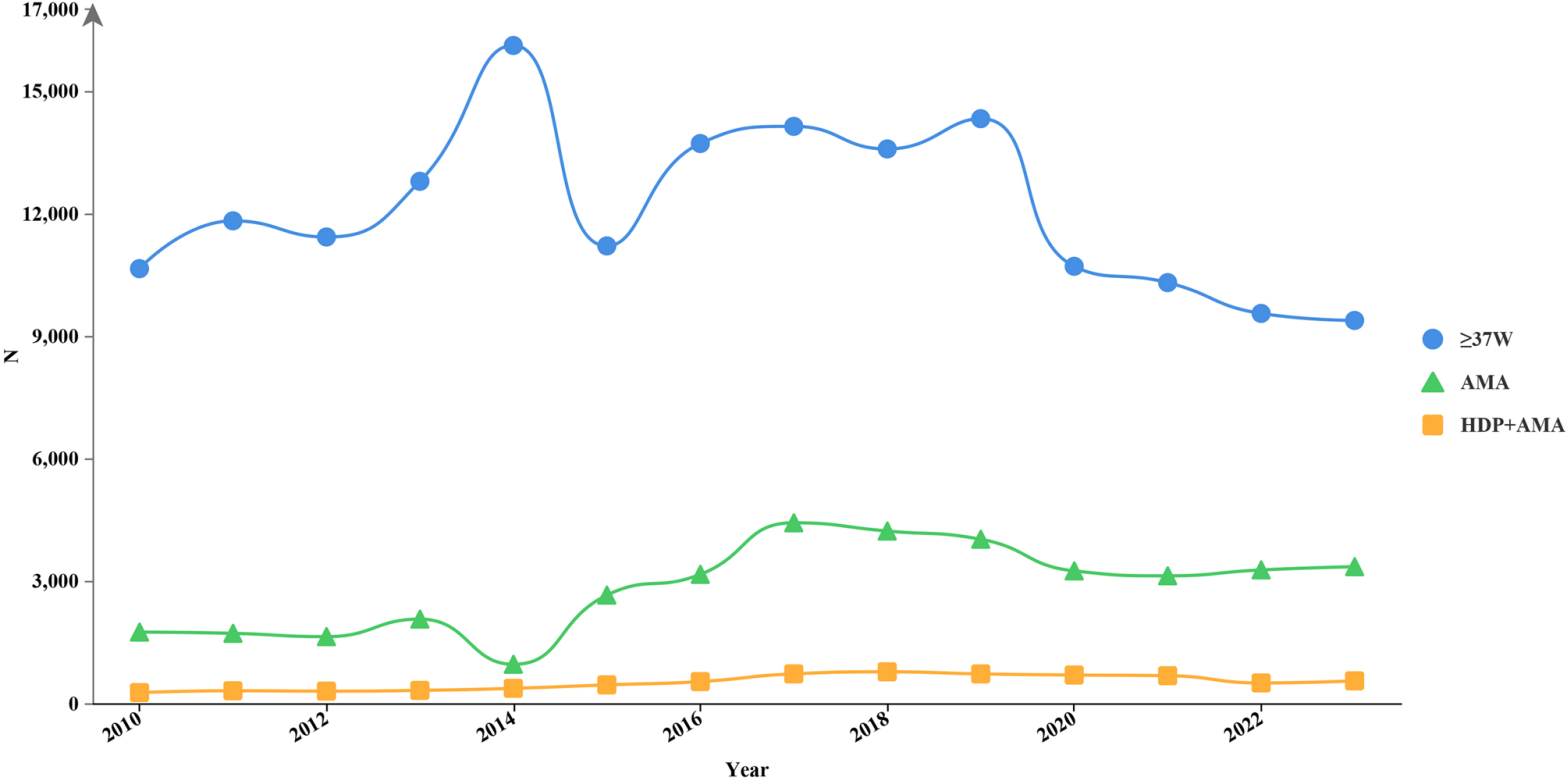
Temporal patterns in obstetric volumes and maternal age distribution (2010–2023). The line chart depicts longitudinal trends across three key obstetric parameters: full-term births (blue line, ≥ 37 weeks’ gestation), AMA deliveries (green line, maternal age of ≥ 35 years), and AMA + HDP (yellow line). The Y-axis ranges 0–17,000 using consistent unit intervals, and the X-axis displays complete obstetric years (2010–2023). Data smoothing was applied using 1-year moving averages. The definition of AMA aligned with the FIGO 2015 criteria

From a screened cohort of 220,907 deliveries, 81,107 term pregnancies were identified, including 2100 cases of HDP managed under the WHO partograph (2010–2014) and 3706 cases of HDP managed under Zhang’s guideline (2014–2023, Fig. 1). The prevalence of HDP increased from 6.7% (2100/31,286) in the 1994 WHO group to 7.4% (3706/49,821) in Zhang’s guideline group. Figure 2 presents the delivery data trends from 2010 to 2023.The number of deliveries by AMA parturients rose rapidly after 2014 and then plateaued. The number of primiparous deliveries involving women with AMA complicated by HDP increased slowly over time. This reflects the effects of fertility intention on the population structure, with the proportion of advanced-aged and high-risk parturients increasing over time. There is a need to optimize the intrapartum management of high-risk pregnancies. Women with HDP managed under Zhang’s guideline were significantly older (31.51 ± 3.71 years vs. 30.40 ± 3.12 years, *P <* 0.0001), and they had higher rates of AMA (20.72% vs. 9.24%, *P <* 0.0001) and ART utilization (6.99% vs. 0.90%, *P <* 0.0001, Table 1).

**Table 1:** Demographic characteristics by partograph group in patients with full-term hypertensive disorder of pregnancy

Variables	Total (*N* = 5806)	^c^1994 WHO (2010–2014.08) (*N* = 2100)	^d^Zhang’s (2014.09–2023) (*N* = 3706)	*P*
Maternal age (years)	31.10 ± 3.55	30.40 ± 3.12	31.51 ± 3.71	< 0.0001
^a^AMA (*n*, %)	962 (16.57)	194 (9.24)	768 (20.72)	< 0.0001
Gestational age at delivery(weeks)	38.96 ± 1.06	38.92 ± 1.07	38.99 ± 1.05	0.0188
Assisted reproduction (*n*, %)	278 (4.79)	19 (0.90)	259 (6.99)	< 0.00001
^b^Pregnancy with obesity (*n*, %)	435 (7.49)	90 (4.29)	345 (9.31)	< 0.0001
Gestational diabetes mellitus (*n*, %)	1227 (21.13)	350 (16.67)	877 (23.66)	< 0.0001

^a^ age≥35 years

^b^defined as BMI≥28 kg/m^2^ in China

^c^labor guided by the 1994 WHO partograph

^d^labor guide by Zhang’s guideline

The women with HDP in Zhang’s guideline cohort exhibited elevated rates of obesity (9.31% vs. 4.29%, *P <* 0.0001) and GDM (23.66% vs. 16.67%, *P <* 0.0001, Table 1). Neonatal outcomes, including birth weight and macrosomia rates, remained comparable between the groups. Temporal stratification of HDP subtypes (Table 2) demonstrated increased rates of chronic hypertension with superimposed preeclampsia and preeclampsia under Zhang’s guideline after 2014, whereas the rates of gestational hypertension and chronic hypertension were decreased in this group.

**Table 2:** Temporal trends in full-term hypertensive disorder of pregnancy classification (2010–2023)

Period	Total(*n*)	Gestational hypertension (*n*, %)	Preeclampsia (*n*, %)	Chronic hypertension (*n*, %)	Chronic hypertension with superimposed preeclampsia (*n*, %)
2010–2014	4702	2514(53.47%)	1340(28.50%)	687(14.61%)	161(3.42%)
2015–2018	3964	1760(44.40%)	1598(40.31%)	372(9.39%)	234(5.90%)
2019–2023	5414	2358(43.55%)	2108(38.94%)	536(9.90%)	412(7.61%)

The classification followed the International Society for the Study of Hypertension in Pregnancy criteria: gestational hypertension, preeclampsia, chronic hypertension, and chronic hypertension with superimposed preeclampsia. Key clinical milestones: 2014 (Zhang’s guideline implementation) and 2018 (International Society for the Study of Hypertension in Pregnancy diagnostic update). 2010–2014: pre-implementation period of Zhang’s labor guideline (control baseline), including the management phase of the WHO partograph.2015–2018: post-implementation period of Zhang’s guideline (since September 2014) until before the update of International Society for the Study of Hypertension in Pregnancy diagnostic criteria (2018 as the key node). 2019–2023: stable period after International Society for the Study of Hypertension in Pregnancy update, allowing observation of the dual effects of the guideline and diagnostic criteria

### Labor interventions and delivery outcomes

**Fig. 3:**
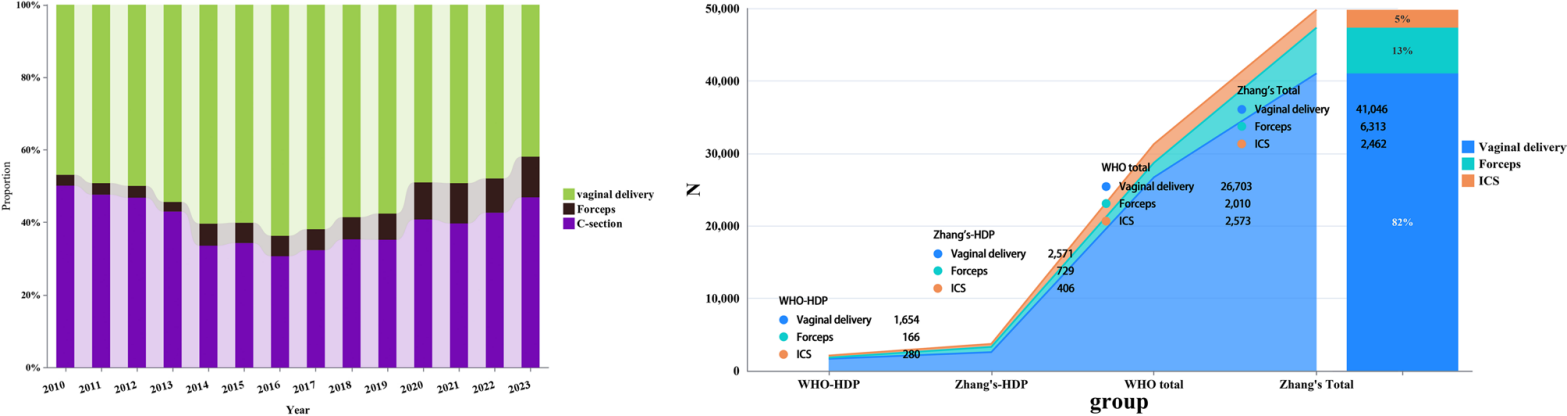
Delivery mode patterns in full-term HDP cohorts. **A** Stacked bar graph presenting the temporal evolution of delivery modes (2010–2023): spontaneous vaginal (green), operative vaginal (brown), and cesarean (purple). Annual proportions are annotated (e.g., 2020: SVD 48.96%, OVD 10.24%, CD 40.8%). The dashed trend line represents the cesarean rate reduction from50.16% (2010) to 30.7% (2016) followed by a gradual increase to 46.94% (2023). **B** Combined line and bar chart comparing the delivery mode distributions between partograph protocols: WHO vs. Zhang’s. Axes represent mode percentages with 10% increments. Key differences in HDP: spontaneous vaginal (WHO 78.8% vs. Zhang’s 69.3%), cesarean (WHO 13.3% vs. Zhang’s 11.0%), forceps-assisted (WHO 7.9% vs. Zhang’s 19.7%). Statistical significance (χ^2^ test) was denoted by *P <* 0.05 or *P < 0.01. All data were derived from the electronic medical records of singleton vertex presentations

Zhang’s guideline was associated with reduced rates of labor interventions (16.06% vs. 43.62%, *P <* 0.0001), including lower rates of oxytocin augmentation (8.72% vs. 13.52%, *P <* 0.0001) and artificial membrane rupture (3.59% vs. 5.43%, *P* = 0.0008), but labor analgesia use was greater under Zhang’s guideline (58.45% vs. 3.48%, *P <* 0.0001, Table 3). Intrapartum cesarean rates declined under Zhang’s guideline (10.96% vs. 13.33%, *P <* 0.0001), with a sustained reduction observed from 2014 to 2023 (Fig. 3a–b). Conversely, forceps-assisted deliveries increased significantly under Zhang’s guideline (19.67% vs. 7.90%, *P <* 0.0001), whereas vaginal delivery rates remained higher in the 1994 WHO group (78.76% vs. 69.37%, *P <* 0.0001).​ The wide spread use of labor analgesia alleviated maternal pain but impaired pushing efficacy, leading to higher forceps delivery rates (odds ratio [OR] = 2.268, Table 4).

**Table 3:** Comparison of labor interventions and obstetrics outcomes between the 1994 WHO partograph and zhang’s guideline groups in full-term hypertension disorder of pregnancy deliveries

Variables	Total (*N* = 5806)	^*^1994 WHO (2010–2014.08) (*N* = 2100)	^**^Zhang’s (2014.09–2023) (*N* = 3706)	*p*
Total duration of labor (h)	10.08 ± 5.19	9.09 ± 4.51	10.67 ± 5.47	< 0.0001
^a^Labor intervention (*n*, %)	1511 (26.02)	916 (43.62)	595 (16.06)	< 0.0001
Oxytocin augmentation (*n*, %)	607 (10.45)	284 (13.52)	323 (8.72)	< 0.0001
Artificial rupture of membrane (*n*, %)	247 (4.25)	114 (5.43)	133 (3.59)	0.0008
Labor analgesia (*n*, %)	2239 (38.56)	73 (3.48)	2166 (58.45)	< 0.0001
^b^Intrapartum fever (*n*, %)	235 (4.05)	25 (1.19)	210 (5.67)	< 0.0001
Lateral episiotomy (*n*, %)	1599 (27.54)	825 (39.29)	774 (20.89)	< 0.0001
Injury of anal sphincter (*n*, %)	2 (0.03)	0	2 (0.05)	0.2870
^c^Eclampsia (*n*, %)	5 (0.086%)	2(0.09)	3(0.08)	0.6422
^d^HELLP syndrome (*n*, %)	26 (0.45%)	3(0.14)	23(0.62)	< 0.0001
Placental abruption (*n*, %)	97 (1.67%)	30(1.42)	67(1.81)	< 0.0001
Birth weight (g)	3308.01 ± 427.63	3318.27 ± 429.14	3302.20 ± 426.72	0.1690
^e^Macrosomia (*n*, %)	322 (5.55)	117 (5.57)	205 (5.53)	0.9492
^f^Low birth weight (*n*, %)	158 (2.72)	59 (2.81)	99 (2.67)	0.7558
Neonatal asphyxia rate (*n*, %)	13 (0.22)	6 (0.29)	7 (0.19)	0.4532
Bleeding volume (mL)	365.00 ± 213.51	323.24 ± 180.87	388.36 ± 226.45	< 0.0001
Postpartum hemorrhage rate (*n*, %)	978 (16.84)	236 (11.24)	742 (20.02)	< 0.0001
Blood transfusion (*n*, %)	197 (3.39)	47 (2.24)	150 (4.05)	0.0003
^h^Postpartumurinary retention (*n*, %)	421 (7.25)	122 (5.81)	299 (8.07)	0.0014
Injury of anal sphincter (*n*, %)	2 (0.03)	0	2 (0.05)	0.2870
Delivery mode				< 0.0001
Vaginal delivery	4225 (72.77)	1654 (78.76)	2571 (69.37)	
Forceps	895 (15.42)	166 (7.90)	729 (19.67)	
Intrapartum cesarean section	686 (11.82)	280 (13.33)	406 (10.96)	

^a^ labor intervention includes oxytocin augmentation, artificial rupture of membrane, and episiotomy

^b^ intrapartum fever refers to fever during labor. Fever is defined as an elevation of body temperature of ≥ 38 °C(100.4 °F)

^c^ eclampsia describes seizures that occur in pregnant women with preeclampsia

^d^ *H* syndrome is characterized by hemolysis, *EL* elevated liver enzymes, and *LP* low platelets in a pregnant or puerperal patient

^e^ macrosomia, birth weight of at least 4,000 g

^f^ low birth weight, weight at birth of 2500 g

^g^neonatalasphyxia,5-min Apgar score < 7

^h^postpartumurinary retention, the absence of spontaneous micturition more than 6 h after birth or when residual volume after urination is less than 150 mL

**Table 4:** Multivariate logistic regression analysis of factors associated with forceps-assisted delivery in full-term hypertensive disorder of pregnancy deliveries

Variables	Beta	Standard error	OR (95% CI)	*P*
Zhang’s vs.1994 WHO	0.4529	0.1098	1.573 (1.268–1.950)	< 0.0001
^a^AMA	0.3741	0.0942	1.454 (1.209–1.749)	< 0.0001
^b^Pregnancy with obesity	−0.0864	0.1409	0.917 (0.696–1.209)	0.5398
Gestational diabetes mellitus	0.1622	0.0911	1.176 (0.984–1.406)	0.0750
Assisted reproduction	0.2027	0.1545	1.225 (0.905–1.658)	0.1896
Labor duration > 20 h	0.6284	0.1619	1.875 (1.365–2.575)	0.0001
Labor analgesia	0.8191	0.0901	2.268 (1.901–2.706)	< 0.0001
Oxytocin augmentation	−0.0835	0.1292	0.920 (0.714–1.185)	0.5182
^c^Macrosomia	0.1425	0.1563	1.153 (0.849–1.566)	0.3617
Gestational age at delivery	0.1554	0.037	1.168 (1.086–1.256)	< 0.0001

*OR* odds ratio, *CI *confidence interval

^a^ age≥35 years

^b^ defined as BMI≥28 kg/m^2^ in China

^c^birth weight≥4000 g

The incidence of PPH was nearly 2-fold higher under Zhang’s guideline (20.02% vs. 11.24%, *P <* 0.0001), accompanied by higher transfusion rates (4.05% vs. 2.24%, *P =* 0.0003). The rates of neonatal asphyxia (0.19% vs. 0.29%, *P =* 0.4532) and severe perineal lacerations (*P =* 0.2870) did not differ significantly between the groups. The duration of labor was prolonged in Zhang’s guideline group (10.67 ± 5.47 h vs. 9.09 ± 4.51 h, *P <* 0.0001). Although Zhang’s guideline was associated with higher PPH rates, the institutional capacity for hemorrhage management (e.g., transfusion rates) was only modestly elevated (4.05% vs. 2.24%, *P* = 0.0003, Table 3).

### Multivariate analysis of risk factors

Multivariable regression identified Zhang’s guideline as an independent risk factor for PPH (OR = 2.101, 95% confidence interval [CI] = 1.766–2.498, *P <* 0.0001), in addition to forceps delivery (OR = 1.573, 95% CI = 1.268–1.950, *P <* 0.0001). AMA and prolonged labor (> 20 h) significantly increased the risks of adverse outcomes (Tables 5 and 4).

**Table 5:** Multivariate logistic regression analysis of risk factors for postpartum hemorrhage in full-term hypertensive disorder of pregnancy deliveries

Variables	Beta	Standard error	OR (95% CI)	*P*
Zhang’s vs.1994 WHO	0.7422	0.0885	2.101 (1.766–2.498)	< 0.0001
^a^AMA	−0.1471	0.1008	0.863 (0.708–1.052)	0.1446
^b^Pregnancy with obesity	−0.2271	0.1455	0.797 (0.599–1.060)	0.1184
Gestational diabetes mellitus	−0.2501	0.0949	0.779 (0.647–0.938)	0.0084
Assisted reproduction	0.2518	0.1548	1.286 (0.950–1.742)	0.1038
Labor duration > 20 h	0.4743	0.1698	1.607 (1.152–2.242)	0.0052
Oxytocin augmentation	0.5224	0.1136	1.686 (1.350–2.106)	< 0.0001
^**c**^Macrosomia	0.7375	0.1332	2.091 (1.610–2.715)	< 0.0001
Episiotomy	0.7285	0.1003	2.072 (1.702–2.522)	< 0.0001
Delivery mode				
Vaginal delivery	Reference		1	
Forceps	1.5529	0.101	4.725 (3.876–5.760)	< 0.0001
Intrapartum cesarean section	1.7958	0.1109	6.024 (4.848–7.486)	< 0.0001

*OR* odds ratio, *CI *confidence interval

^a^ age ≥ 35 years

^b^ defined as BMI ≥ 28 kg/m^2^ in China

^c^ birth weight ≥ 4000 g

The observed intergroup differences stemmed from a combination of demographic shifts (AMA, ART, comorbidities), protocol modifications (redefinition of active labor, expanded analgesia utilization), and diagnostic updates (ISSHP criteria). Collectively, these factors, together with improved fetal monitoring, enhanced clinician expertise, and institutional and technological advances, contributed to the outcomes through their interactions with guideline adherence.

## Discussion

The rising incidence of HDP reflects global shifts in lifestyle factors (e.g., obesity) and demographic trends (e.g., AMA) [22], necessitating enhanced strategies for HDP prevention and management [23].Timely delivery remains a cornerstone of HDP management, but clinical practices vary widely. Studies indicated that once at or beyond 37 weeks’ gestation, delivery is recommended on the basis of the HYPITAT findings [24]. However, health professionals often fear that vaginal delivery will worsen the maternal and fetal condition [25]. Zhang’s guideline addresses this issue by simplifying labor dystocia criteria and redefining the active phase onset at 6 cm of dilation, thereby promoting physiological labor and reducing cardiovascular stress, particularly in nulliparous women [26]. The application of labor analgesia in patients with full-term HDP ensures adequate pain relief during childbirth, thereby alleviating the increase in blood pressure that can occur during labor and increasing the success rate of spontaneous vaginal delivery [27].

This study highlighted key differences between Zhang’s guideline and the 1994 WHO partograph in managing HDP. Overall, Zhang’s guideline displayed certain advantages in reducing labor intervention and reducing the rate of ICS. However, the guideline was associated with increased forceps usage and a higher incidence of PPH.

### Factors contributing to lower ICS rates under Zhang’s guideline

Despite certain controversies regarding different intrapartum management models, the promotion of vaginal delivery and reduction of cesarean section rates remain the core objectives of intrapartum care while ensuring maternal and fetal safety. A multicenter randomized controlled trial published in *Lancet* in 2019 revealed that ICS rates remained consistent across different labor standards, with emphasis on labor progression identified as the key factor for reducing ICS rates [28].Within this study, Zhang’s guideline significantly lowered ICS rates among women with HDP. The primary surgical indications for ICS under Zhang’s guideline were fetal distress (31.4%), relative cephalopelvic disproportion (11.58%), and intrapartum fever (11.08%).

Four key factors might have contributed to the reduced ICS rate in Zhang’s group. First, the decreased use of oxytocin augmentation and artificial rupture of membranes minimized iatrogenic risks of fetal distress or abnormal labor progression. Second, widespread labor analgesia improved maternal psychological states, reducing cesarean sections necessitated by labor arrest from pain or maternal intolerance. Third, flexible labor management including extended latent and active phases (using a 6-cm dilation threshold) avoided premature interventions, whereas optimized management of high-risk pregnancies (e.g., AMA, gestational diabetes, obesity) addressed comorbidities linked to elevated cesarean rates (e.g., 40% in obese women) [29]. Fourth, higher forceps use reduced the risk of second-stage ICS, providing a safe alternative for delivery completion. This strategy provided an effective alternative while ensuring maternal and fetal safety. Furthermore, advancements during the study period (2010–2023), such as improved continuous electronic fetal heart rate monitoring and clinician training, enhanced intrapartum observation. Notably, the ICS rate attributable to fever was higher in Zhang’s group. Under this protocol, patients with extended labor durations with prolonged oxytocin exposure carried an increased intrapartum fever risk [30]. Overall, the decreased ICS rate in Zhang’s group resulted from the combined effects of reduced interventions, widespread labor analgesia, optimized high-risk pregnancy management, and the substitute effect of forceps-assisted delivery.

### Comparative analysis of PPH risk in patients with HDP: Zhang’s guideline vs. 1994 WHO partograph

Zhang’s guideline was associated with higher PPH rates. Multivariate regression identified Zhang’s protocol, gestational diabetes, oxytocin use, macrosomia, episiotomy, labor duration of >20 h, and higher rates of forceps delivery or cesarean conversion as independent PPH risk factors. These findings align with prior evidence linking prolonged labor (>20 h) and oxytocin administration to PPH risk in nulliparous patients with HDP and vaginal deliveries [31–33]. The association persisted even after adjusting for confounders, with oxytocin-induced desensitization of uterine receptors potentially impairing postpartum contractions. Whereas AMA and obesity were associated with a lower PPH risk than reported elsewhere, forceps-assisted delivery and ICS were confirmed as significant contributors. Additionally, oxytocin and epidural anesthesia were correlated with prolonged second-stage labor, elevating operative delivery risks and the risk of subsequent PPH [34]. Preeclampsia further exacerbates PPH susceptibility in vaginal deliveries because of underlying features such as endothelial dysfunction, abnormal uteroplacental perfusion, and coagulation defects, although these might be absent in mild cases [35, 36].

Key contributors to elevated PPH rates under Zhang’s guideline included prolonged labor (which exacerbated uterine atony), a higher rate of forceps-related trauma, increased analgesia usage (potentially impairing uterine contraction efficiency) and a higher incidence of HELLP syndrome and placental abruption (which further amplified hemorrhagic risks). Multivariate analysis confirmed Zhang’s protocol as an independent risk factor for PPH. These findings underscore the need for individualized labor strategies in HDP populations to mitigate PPH and transfusion risks.

### Increased forceps use in patients with HDP under Zhang’s guideline

Zhang’s labor management guideline was associated with a significantly higher rate of forceps-assisted deliveries in patients with HDP, in line with global trends. In the UK, forceps use accounts for more than 50% of operative vaginal deliveries (OVDs; refers to the use of an instrument [forceps or vacuum device]to assist with the delivery of the fetus from the vagina), with OVD rates ranging from 5.4% to 20% [37], whereas an OVD rate of 8.4% was reported in Japan [38]. The indications for forceps use, including prolonged second-stage labor, fetal distress, abnormal fetal position, and maternal conditions requiring expedited delivery [39], reflect efforts to reduce second-stage cesarean sections and associated morbidity, consistent with our findings [40].

The elevated utilization of forceps under Zhang’s guideline, primarily attributed to prolonged labor duration, increased the risk of fetal distress and consequently necessitated instrumental delivery assistance. Second, clinicians might prioritize forceps over cesarean sections in the second stage of labor in patients with HDP, particularly given the heightened surgical risks in this population. Third, the higher analgesia utilization rate under Zhang’s protocol might have impaired pushing efficacy. Fourth, endothelial dysfunction and placental insufficiency in patients with HDP heighten susceptibility to fetal distress, prompting clinicians to expedite delivery *via* forceps to mitigate hypoxia risks. Although OVD can reduce cesarean-related morbidity, the elevated forceps use under Zhang’s protocol highlights potential trade-offs between intervention thresholds and birth outcomes. Individualized management, incorporating dynamic risk assessment of fetal status, maternal comorbidities, and labor progression, might optimize delivery mode selection and minimize unnecessary instrumental interventions.

### Increased use of labor analgesia in Zhang’s guideline

The use of labor analgesia was significantly higher for Zhang’s guideline (58.45%) than for the 1994 WHO partograph (3.48%), and this difference had a dual impact on delivery outcomes. On the positive side, analgesia helped relieve pain and anxiety, thereby reducing cases of labor arrest caused by pain or psychological stress. This indirectly contributed to a lower intrapartum cesarean rate in Zhang’s guideline group, which aligns with the guideline’s goal of reducing unnecessary interventions. Additionally, it lessened pain-related surges in blood pressure among patients with HDP, supporting better management of HDP.

On the risk side, analgesia also led to a series of cascading effects. Neuraxial analgesia reduced pushing efficacy, and when combined with the extended labor observation thresholds under Zhang’s guideline, it prolonged the total labor duration and increased the rate of forceps-assisted delivery. Multivariate analysis confirmed that analgesia was an independent factor associated with increased forceps use (OR = 2.268, *P* < 0.0001). Furthermore, analgesia-related prolongation of labor, together with trauma from forceps delivery, might have contributed to the higher rate of PPH observed in Zhang’s guideline group (20.02% vs. 11.24%). It should be noted that prolonged labor itself is an independent risk factor for PPH (OR = 1.607, *P* = 0.0052). Analgesia might also have been associated with a higher incidence of intrapartum fever in Zhang’s group (5.67% vs. 1.19%), which is likely attributable to mild inflammation or epidural-related factors, although most cases were mild.

Particular caution is warranted regarding the use of neuraxial analgesia in women with thrombocytopenia or other hematologic complications, which are common in patients with HDP. For patients with HDP without definite contraindications but with high-risk features, labor analgesia should be administered cautiously under close monitoring of blood pressure, coagulation function, and fetal heart rate, with emergency preparedness undertaken to weigh the benefits of analgesia against its potential risks.

### Neonatal asphyxia rates under Zhang’s guideline and the 1994 WHO partograph

Neonatal asphyxia rates did not significantly differ between Zhang’s guideline and the 1994 WHO partograph despite variations in clinical management. Three factors might explain this finding. First, Zhang’s protocol featured markedly lower intrapartum intervention rates, including reduced use of artificial rupture of membranes and oxytocin induction. Second, although fewer interventions might mitigate procedure-related fetal distress, the minimal asphyxia rate difference suggests limited clinical impact. Third, although analgesia alleviates maternal stress and it can indirectly improve fetal oxygenation, its direct effect on neonatal outcomes remains unclear. Despite the larger sample size of Zhang’s guidelines cohort (3706 vs. 2100), the absolute number of cases of asphyxia differed by only one (seven vs. six), likely reflecting insufficient statistical power rather than true clinical variation. These results imply that Zhang’s reduced-intervention approach balances procedural risks with neonatal safety, though larger studies are needed for confirmation.

### AMA and ART utilization in HDP

Zhang’s guideline was applied in a higher proportion of AMA pregnancies and ART conceptions than the 1994 WHO protocol. Four interrelated factors potentially drove this disparity. First, data from Zhang’s cohort (2014–2023) reflect China’s delayed childbearing norms, increasing the prevalence of AMA. Second, declining fertility in AMA populations has heightened reliance on ART, which Zhang’s guideline accommodates through optimized monitoring and reduced interventions. Third, the higher rates of obesity and gestational diabetes in Zhang’s group align with AMA’s role as a risk factor for these conditions [41, 42]. Finally, the emphasis of Zhang’s guideline on low-intervention strategies and analgesia might enhance compliance in high-risk AMA/ART pregnancies. However, the elevated PPH and forceps rates underscore the need to balance reduced interventions with maternal safety, particularly given ART’s association with HDP risk (OR = 1.71) [43–45].

### Strengths and limitations

This study’s strengths and limitations reflect the challenges in evaluating guideline implementation in real-world obstetric practice. A key strength lies in its larger, decade-spanning sample size, enabling robust analysis of delivery mode trends and HDP outcomes under Zhang’s guideline. However, methodological constraints require acknowledgment. First, the retrospective design introduced potential information biases from medical record reviews, compounded by reliance on data from a single tertiary hospital in Northern China. Unmeasured confounders, such as institutional variations in clinical protocols, documentation practices, and provider decision-making, might have influenced the observed associations between partograph use and outcomes. Second, the single-center nature of the study limited its generalizability, as regional differences in obstetric practices and patient demographics (e.g., socioeconomic factors, comorbid profiles) might reduce external validity. Third, the study focused on nulliparous women, and factors present in multiparous women, such as uterine contractility patterns and birth canal resilience, could influence the applicability of Zhang’s guideline. Fourth, because this study was conducted over a long period, the methods for managing PPH might have changed over time. These changes could involve new or more frequent use of treatments such as intrauterine balloon tamponade, changes in the type or dose of medicines used to control bleeding, or updated blood transfusion practices. Because our study did not track or account for these variations, they might have influenced the PPH outcomes over time. For example, the higher PPH rate observed in Zhang’s guideline group could be partly attributable to these changes rather than the guideline itself. This would thus affect the interpretation of differences in PPH results between the two management protocols. Fifth, the clinical decision to perform episiotomy might have influenced the overall labor intervention rate. Although our department followed a standardized protocol and maintained oversight of the attending clinicians’ decision-making quality, episiotomy decisions ultimately incorporated a degree of individual discretion, introducing inherent subjectivity. This variability in clinicians’ judgment of indications was not quantified or controlled in our retrospective analysis, and this might have confounded the interpretation of differences in labor intervention rates between the two protocols. Future research assessing the efficacy of Zhang’s guideline in multiparous women, particularly regarding potential risks of PPH or instrumental delivery with prolonged labor observation, would be warranted to refine its clinical utility among diverse obstetric populations.

## Conclusions

Compared with the 1994 WHO partograph, Zhang’s guideline for managing HDP demonstrated effectiveness in reducing ICS and labor interventions. It also proved more adaptable to pregnancies involving AMA and ART without increasing the risk of neonatal asphyxia. However, its implementation was associated with higher rates of PPH and forceps delivery. Notably, the protocol itself emerged as an independent risk factor for PPH. These findings highlight the need for risk-stratified, individualized labor management strategies to optimize maternal and fetal outcomes in hypertensive disorders of pregnancy.

## Data Availability

The datasets used and/or analyzed during the current study are available from the corresponding author on reasonable request.

## Abbreviations

HDP: Hypertensive disorder of pregnancy
AMA: Advanced maternal age
ART: Assisted reproductive technology
ICS: Intrapartum cesarean section
PPH: Postpartum hemorrhage
ISSHP: International Society for the Study of Hypertension in Pregnancy
BMI: Body mass index
OVDs: Operative vaginal deliveries

## References

[1] Ong KL, Stafford LK, McLaughlin SA, Boyko EJ, Vollset SE, Smith AE, et al. Global, regional, and national burden of diabetes from 1990 to 2021, with projections of prevalence to 2050: a systematic analysis for the global burden of disease study 2021. Lancet. 2023;402(10397):203–34. 10.1016/S0140-6736(23)01301-6. 37356446 10.1016/S0140-6736(23)01301-6 PMC10364581

[2] Magee LA, Brown MA, Hall DR, Gupte S, Hennessy A, Karumanchi SA, et al. The 2021 international society for the study of hypertension in pregnancy classification, diagnosis & management recommendations for international practice. Pregnancy Hypertens. 2022;27:148–69. 10.1016/j.preghy.2021.09.008. 35066406 10.1016/j.preghy.2021.09.008

[3] Cruz MO, Gao W, Hibbard JU. What is the optimal time for delivery in women with gestational hypertension?Am. J Obstet Gynecol. 2012;207(3):214. 10.1016/j.ajog.2012.06.009. .e1-6. 10.1016/j.ajog.2012.06.009 22831812

[4] Magee LA, Tohill S, Kirkham K, Evans R, Gkini E, Moakes CA, et al. WILL (when to induce labour to limit risk in pregnancy hypertension): a multicentre randomised controlled trial - adaptations to deliver a timing-of-birth trial during the COVID-19 international pandemic. Trials. 2022;23(1):884. 10.1186/s13063-022-06834-4. 36271441 10.1186/s13063-022-06834-4 PMC9585855

[5] Metoki H, Iwama N, Hamada H, Satoh M, Murakami T, Ishikuro M, et al. Hypertensive disorders of pregnancy: definition, management, and out-of-office blood pressure measurement. Hypertens Res. 2022;45(8):1298–309. 10.1038/s41440-022-00965-6. 35726086 10.1038/s41440-022-00965-6 PMC9207424

[6] World Health Organization partograph in management of labour. World Health Organ Maternal Health Safe Mother Programme Lancet. 1994;343(8910):1399–404. 7910888

[7] Lavender T, Cuthbert A, Smyth RM. Effect of partograph use on outcomes for women in spontaneous labour at term and their babies. Cochrane Database Syst Rev. 2018;8(8):Cd005461. 10.1002/14651858.CD005461.pub5. 30080256 10.1002/14651858.CD005461.pub5 PMC6513424

[8] WHO Guidelines Approved by the Guidelines Review Committee. WHO recommendations: intrapartum care for a positive childbirth experience. Geneva: World Health Organization; 2018. 30070803

[9] Fitzpatrick KE, Tuffnell D, Kurinczuk JJ, Knight M. Pregnancy at very advanced maternal age: a UK population-based cohort study. Bjog. 2017;124(7):1097–106. 10.1111/1471-0528.14269. 27581343 10.1111/1471-0528.14269 PMC5484369

[10] Kramer MS, Berg C, Abenhaim H, Dahhou M, Rouleau J, Mehrabadi A, Joseph KS. Incidence, risk factors, and Temporal trends in severe postpartum hemorrhage. Am J Obstet Gynecol. 2013;209(5):449. 10.1016/j.ajog.2013.07.007. .e1-7. 10.1016/j.ajog.2013.07.007 23871950

[11] Zhang J, Landy HJ, Ware Branch D, Burkman R, Haberman S, Gregory KD, et al. Contemporary patterns of spontaneous labor with normal neonatal outcomes. Obstet Gynecol. 2010;116(6):1281–7. 10.1097/AOG.0b013e3181fdef6e. 21099592 10.1097/AOG.0b013e3181fdef6e PMC3660040

[12] Sun C, Su S, Song W, Jiang H. Use of the WHO partograph and zhang’s guideline for labor and delivery in China: implications for clinical practice. BMC Pregnancy Childbirth. 2024;24(1):799. 10.1186/s12884-024-06985-z. 39604879 10.1186/s12884-024-06985-z PMC11600908

[13] Jovanovski AP. Randomized controlled trial of prolonged second stage: extending the time limit vs usual guidelines. Am J Obstet Gynecol. 2016;215(4):535. 10.1016/j.ajog.2016.05.043. 27262974 10.1016/j.ajog.2016.05.043

[14] Rosenbloom JI, Stout MJ, Tuuli MG, Woolfolk CL, López JD, Macones GA, Cahill AG. New labor management guidelines and changes in Cesarean delivery patterns. Am J Obstet Gynecol. 2017;217(6):689. 10.1016/j.ajog.2017.10.007. 10.1016/j.ajog.2017.10.007 PMC5712240 29037483

[15] Kadour-Peero E, Sagi S, Awad J, Bleicher I, Gonen R, Vitner D. Are we preventing the primary Cesarean delivery at the second stage of labor following ACOG-SMFM new guidelines? Retrospective cohort study. J Matern Fetal Neonatal Med. 2022;35(25):6708–13. 10.1080/14767058.2021.1920913. 33980117 10.1080/14767058.2021.1920913

[16] Dalbye R, Blix E, Frøslie KF, Zhang J, Eggebø TM, Olsen IC, et al. The labour progression study (LaPS): duration of labour following Zhang’s guideline and the WHO partograph - a cluster randomised trial. Midwifery. 2020;81:102578. 10.1016/j.midw.2019.102578. 31783231 10.1016/j.midw.2019.102578

[17] Ushida T, Kotani T, Imai K, Nakano-Kobayashi T, Iitani Y, Nakamura N, et al. Prevalence and risk factors of labor-onset hypertension: a multicenter study in Japan. Pregnancy Hypertens. 2021;26:48–53. 10.1016/j.preghy.2021.08.118. 34508948 10.1016/j.preghy.2021.08.118

[18] Lavender T, Bernitz S. Use of the partograph - current thinking. Best Pract Res Clin Obstet Gynaecol. 2020;67:33–43. 10.1016/j.bpobgyn.2020.03.010. 32321672 10.1016/j.bpobgyn.2020.03.010

[19] The expert consensus of new standard and management of. labor(2014): interpretation and statement]. Zhonghua Fu Chan Ke Za Zhi. 2018;53(2):143–4. 10.3760/cma.j.issn.0529-567X.2018.02.020. 10.3760/cma.j.issn.0529-567X.2018.02.020 29534380

[20] Ackerman CM, Platner MH, Spatz ES, Illuzzi JL, Xu X, Campbell KH, et al. Severe cardiovascular morbidity in women with hypertensive diseases during delivery hospitalization. Am J Obstet Gynecol. 2019;220(6):582. 10.1016/j.ajog.2019.02.010. 10.1016/j.ajog.2019.02.010 30742823

[21] National Health and Family Planning Commission of the People’s Republic of China. Criteria of weight for adults. In., vol. WS/T 428 – 2013. Beijing: China Standards; 2013.

[22] Hesketh T, Zhou X, Wang Y. The end of the one-child policy: lasting implications for China. JAMA. 2015;314(24):2619–20. 10.1001/jama.2015.16279. 26545258 10.1001/jama.2015.16279

[23] Li F, Qin J, Zhang S, Chen L. Prevalence of hypertensive disorders in pregnancy in China: a systematic review and meta-analysis. Pregnancy Hypertens. 2021;24:13–21. 10.1016/j.preghy.2021.02.001. 33626437 10.1016/j.preghy.2021.02.001

[24] Koopmans CM, Bijlenga D, Groen H, Vijgen SM, Aarnoudse JG, Bekedam DJ, et al. Induction of labour versus expectant monitoring for gestational hypertension or mild pre-eclampsia after 36 weeks’ gestation (HYPITAT): a multicentre, open-label randomised controlled trial. Lancet. 2009;374(9694):979–88. 10.1016/s0140-6736(09)60736-4. 19656558 10.1016/S0140-6736(09)60736-4

[25] Zhou Y, Yin S, Sheng Q, Yang J, Liu J, Li H, et al. Association of maternal age with adverse pregnancy outcomes: a prospective multicenter cohort study in China. J Glob Health. 2023;13:04161. 10.7189/jogh.13.04161. 38038697 10.7189/jogh.13.04161 PMC10691438

[26] Lee N, Flynn J, Gao Y, Kildea S. Comparing compliance with commencement and use of two partograph designs for women in active labour: A randomised controlled trial. Women Birth. 2023;36(1):e17–24. 10.1016/j.wombi.2022.04.004. 35400605 10.1016/j.wombi.2022.04.004

[27] Han B, Xu M. Effect of continuous spinal anesthesia on the hemodynamics of labor analgesia in hypertensive pregnant women: a comparative, randomized clinical trial. BMC Anesthesiol. 2023;23(1):205. 10.1186/s12871-023-02174-1. 37312032 10.1186/s12871-023-02174-1 PMC10262455

[28] Bernitz S, Dalbye R, Zhang J, Eggebø TM, Frøslie KF, Olsen IC, et al. The frequency of intrapartum caesarean section use with the WHO partograph versus zhang’s guideline in the labour progression study (LaPS): a multicentre, cluster-randomised controlled trial. Lancet. 2019;393(10169):340–8. 10.1016/s0140-6736(18)31991-3. 30581039 10.1016/S0140-6736(18)31991-3

[29] Subramaniam A, Jauk VC, Goss AR, Alvarez MD, Reese C, Edwards RK. Mode of delivery in women with class III obesity: planned Cesarean compared with induction of labor. Am J Obstet Gynecol. 2014;211(6):700. 10.1016/j.ajog.2014.06.045. .e1-9. 10.1016/j.ajog.2014.06.045 24956550

[30] Wang D, Ye S, Tao L, Wang Y. The impact of a new standard labor protocol on maternal and neonatal outcomes. Arch Gynecol Obstet. 2017;296(6):1085–90. 10.1007/s00404-017-4536-0. 28948341 10.1007/s00404-017-4536-0

[31] Huang CR, Xue B, Gao Y, Yue SW, Redding SR, Wang R, et al. Incidence and risk factors for postpartum hemorrhage after vaginal delivery: a systematic review and meta-analysis. J Obstet Gynaecol Res. 2023;49(7):1663–76. 10.1111/jog.15654. 37069822 10.1111/jog.15654

[32] Davey MA, Flood M, Pollock W, Cullinane F, McDonald S. Risk factors for severe postpartum haemorrhage: a population-based retrospective cohort study. Aust N Z J Obstet Gynaecol. 2020;60(4):522–32. 10.1111/ajo.13099. 31758550 10.1111/ajo.13099

[33] Bernitz S, Betran AP, Gunnes N, Zhang J, Blix E, Øian P, et al. Association of oxytocin augmentation and duration of labour with postpartum haemorrhage: a cohort study of nulliparous women. Midwifery. 2023;123:103705. 10.1016/j.midw.2023.103705. 37244235 10.1016/j.midw.2023.103705

[34] Pergialiotis V, Bellos I, Antsaklis A, Papapanagiotou A, Loutradis D, Daskalakis G. Maternal and neonatal outcomes following a prolonged second stage of labor: a meta-analysis of observational studies. Eur J Obstet Gynecol Reprod Biol. 2020;252:62–9. 10.1016/j.ejogrb.2020.06.018. 32570187 10.1016/j.ejogrb.2020.06.018

[35] Li S, Gao J, Liu J, Hu J, Chen X, He J, et al. Incidence and risk factors of postpartum hemorrhage in China: a multicenter retrospective study. Front Med. 2021;8:673500. 10.3389/fmed.2021.673500. 10.3389/fmed.2021.673500 PMC8419315 34497812

[36] Mol BWJ, Roberts CT, Thangaratinam S, Magee LA, de Groot CJM, Hofmeyr GJ. Pre-eclampsia. Lancet. 2016;387(10022):999–1011. 10.1016/s0140-6736(15)00070-7. 26342729 10.1016/S0140-6736(15)00070-7

[37] Scotland PH. Scottish Pregnancy, Births and Neonatal Data Dashboard. In. Edinburgh. UK; 2025.

[38] Sugai S, Mori Y, Yamawaki K, Shima E, Matsushita M, Yoshihara K, et al. Temporal trends and regional variations in operative vaginal deliveries in Japan: a national representative cohort study. J Obstet Gynaecol Res. 2024;50(9):1494–500. 10.1111/jog.16039. 39082381 10.1111/jog.16039

[39] Qiufeng L, Jie X, Xiahong W, Lixin Y. Changes and analysis of transvaginal forceps delivery rate in primary hospitals in the past 10 years. Ginekol Pol. 2019;90(12):711–6. 10.5603/gp.2019.0122. 31909465 10.5603/GP.2019.0122

[40] Vannevel V, Swanepoel C, Pattinson RC. Global perspectives on operative vaginal deliveries. Best Pract Res Clin Obstet Gynaecol. 2019;56:107–13. 10.1016/j.bpobgyn.2018.09.004. 30392949 10.1016/j.bpobgyn.2018.09.004

[41] Ayala-Ramírez P, Serrano N, Barrera V, Bejarano JP, Silva JL, Martínez R, et al. Risk factors and fetal outcomes for preeclampsia in a Colombian cohort. Heliyon. 2020;6(9):e05079. 10.1016/j.heliyon.2020.e05079. 33015399 10.1016/j.heliyon.2020.e05079 PMC7522495

[42] Sheen JJ, Wright JD, Goffman D, Kern-Goldberger AR, Booker W, Siddiq Z, et al. Maternal age and risk for adverse outcomes. Am J Obstet Gynecol. 2018;219(4):390. 10.1016/j.ajog.2018.08.034. 10.1016/j.ajog.2018.08.034 30153431

[43] Chih HJ, Elias FTS, Gaudet L, Velez MP. Assisted reproductive technology and hypertensive disorders of pregnancy: systematic review and meta-analyses. BMC Pregnancy Childbirth. 2021;21(1):449. 10.1186/s12884-021-03938-8. 34182957 10.1186/s12884-021-03938-8 PMC8240295

[44] Almasi-Hashiani A, Omani-Samani R, Mohammadi M, Amini P, Navid B, Alizadeh A, et al. Assisted reproductive technology and the risk of preeclampsia: an updated systematic review and meta-analysis. BMC Pregnancy Childbirth. 2019;19(1):149. 10.1186/s12884-019-2291-x. 31046710 10.1186/s12884-019-2291-x PMC6498659

[45] Moramezi F, Nikbakht R, Saadati N, Farhadi E, Raad N. Comparing the occurrence rate of gestational hypertension during pregnancy with frozen embryo transfer and natural pregnancy. J Fam Med Prim Care. 2023;12(12):3312–8. 10.4103/jfmpc.jfmpc_2429_22. 10.4103/jfmpc.jfmpc_2429_22 PMC10866262 38361845

